# Expert Clinical Management of Severe Immune-Related Adverse Events: Results from a Multicenter Survey on Hot Topics for Management

**DOI:** 10.3390/jcm11205977

**Published:** 2022-10-11

**Authors:** Mar Riveiro-Barciela, Maria Jose Soler, Ana Barreira-Diaz, Sheila Bermejo, Sebastian Bruera, Maria E. Suarez-Almazor

**Affiliations:** 1 Liver Unit, Internal Medicine Department, Vall d’Hebron University Hospital, Vall d’Hebron Barcelona Hospital Campus, 08035 Barcelona, Spain; 2 Centro de Investigación Biomédica en Red de Enfermedades Hepáticas y Digestivas (CIBERehd), Instituto de Salud Carlos III, 28029 Madrid, Spain; 3 Department of Medicine, Autonomous Univeristy of Barcelona (AUB), 08035 Barcelona, Spain; 4 Department of Nephrology, Vall d´Hebron University Hospital, Vall d’Hebron Barcelona Hospital Campus, 08035 Barcelona, Spain; 5 Section of Immunology, Allergy and Rheumatology, Baylor College of Medicine, Houston, TX 77030, USA; 6 Department of Health Services Research and Section of Rheumatology and Clinical Immunology, The University of Texas MD Anderson Cancer Center, Houston, TX 77030, USA

**Keywords:** immune checkpoint inhibitors, immunotherapy, immune-related hepatitis, acute kidney injury, myositis, myocarditis

## Abstract

There are differences in recommendations for the management of immune-related adverse events (irAEs) associated with immune checkpoint inhibitors (ICIs). To assess the real-world management of irAEs, three surveys regarding ICI-induced hepatitis (IIH), renal irAEs, and myositis were developed and sent to experts in each area. Fifty-six surveys were completed (17 IIH, 20 renal irAEs, and 19 myositis). All experts agreed on performing imaging in every suspected case of severe IIH. Sixty-five percent agreed on performing a liver biopsy in patients not responding to corticosteroids. The most common indication for corticosteroid use (59%) was for severe IIH not improving after discontinuation of ICIs. Additionally, 60% of the experts agreed on performing a biopsy for stage 2/3 acute kidney injury (AKI), and 70% recommended imaging for any stage of AKI. Thirty-five percent favored corticosteroids in AKI patients with creatinine levels 2–3-fold above baseline. For myositis, 58% would recommend a muscle biopsy in a patient with weakness and creatine kinase levels of 5000 U/L; 47% would also opt for an endomyocardial biopsy when the troponin levels are increased. Fifty-eight percent recommended oral corticosteroids for myositis, and 37% recommended additional therapy, mainly immunoglobulins. These results show substantial differences in expert practice patterns for the management of severe liver, kidney, and muscular irAEs.

## 1. Introduction

Since the approval of ipilimumab for the treatment of metastatic melanoma in 2010, cancer therapy with immune checkpoint inhibitors (ICIs) targeting either the cytotoxic T-lymphocyte antigen 4 (CTLA-4) or the programmed cell death protein 1 (PD-1) pathway has increased exponentially [1]. There are numerous approved indications for ICI therapy across many cancers [2]. The widespread use of ICIs has resulted in a marked increase in the incidence of immune-related adverse events (irAEs) [3]. The clinical presentations, severity, and prognosis for these irAEs in clinical practice are broad. Therefore, the implementation of general guideline recommendations for irAE management for individual patients may be challenging, emphasizing the importance of the multidisciplinary management of irAEs in these patients [4,5]. To date, the prospective and comparative data on important issues concerning the diagnosis and treatment of specific irAEs are scarce, which can result in variations in practice even among experts. Herein we summarize the results of an international survey of experts on irAEs in gastroenterology, nephrology, and rheumatology inquiring about diagnostic and treatment issues in the management of severe ICI-induced hepatitis (IIH), renal irAEs, and myositis. Acute kidney injury and hepatitis are two of the most common adverse events associated with ICIs, especially when combined therapy with both and anti-PD1 and an anti-CTLA-4 are used [6]. The importance of myositis lies in its potential severity, especially in case of concomitant myocarditis, with high mortality and morbidity rates [7]. Our aim was to assess the real-world management of immune-related hepatitis, acute kidney injury, and myositis caused by ICIs through practice pattern surveys of experts in the field for each of these conditions.

## 2. Materials and Methods

### 2.1. Study Design

Three different surveys were developed for the conditions of interest: IIH, renal irAEs, and myositis. The surveys consisted of six to eight multiple choice and open-ended questions about diagnostic and therapeutic issues related to these irAEs. The main aim of this study was to assess the real-world practice regarding some of the most controversial issues on the management of ICI-induced adverse events. The three surveys are available in the Appendix A.

### 2.2. Participants

Survey participants for each of the three areas of interest were selected by three authors (IIH by M.R.-B., renal irAEs by M.J.S., and myositis by M.S.-A.). Participants were selected if they met the following criteria: active practice in patients with irAEs and expertise based on the authorship of at least 1 published article on the topic of interest. In the case of myositis, the contacted experts were members of either the OMERACT (Outcome Measures in Rheumatology) irAE initiative or a newly formed consortium in the United States investigating irAEs. In the case of acute kidney injury, the contacted experts were members of the ICI-Acute Kidney Injury (AKI) Consortium Investigators. All participants were physicians selected on the basis of their knowledge and clinical practice in each of the three topics and their academic credentials in these areas given their publications and participation in research activities related to irAEs. The surveys were developed by the authors on the basis of topics considered to be most controversial and clinically relevant for the diagnosis and treatment of these irAEs. They also included questions related to the experts’ practices, including their country of practice, years in practice, academic affiliation with a university or medical school, and estimated number of patients with irAEs managed per year.

The surveys and corresponding responses were sent by email. The data are presented in aggregate form; thus, responses cannot be attributed to individual physicians. This study was conducted in compliance with the principles of the Declaration of Helsinki. All authors reviewed and approved the final version of the manuscript.

### 2.3. Statistical Analysis

The analysis was descriptive, primarily reporting frequencies and percentages. Data management and analyses were performed using SPSS software (version 26.0; IBM, Armonk, NY, USA) and figures using Microsoft Excel.

## 3. Results

### 3.1. Management of Severe IIH (Grade 3 or 4)

#### 3.1.1. Participants

We sent out IIH 25 surveys, 17 (68%) of which were completed and returned. The characteristics of the experts who returned the surveys are summarized in Table 1. All of the participants worked in institutions affiliated with universities, with a median time of practice of 10–20 years. Their estimated number of IIH patient visits per year was 20–30, with a third of the experts providing care for at least 30 patients with IIH every year. Regarding location, the highest number of respondents were from Spain, followed by Italy and France.

#### 3.1.2. Diagnosis

All of the experts unanimously agreed on the need to perform imaging in every suspected case of severe IIH. Agreement on the need for ruling out hepatitis E virus (HEV) infection was not universal, although the majority agreed with screening (15/17 [88%]). The most common method of choice for evaluating acute HEV infection was the use of both anti-HEV immunoglobulin (Ig) M and HEV-RNA (10/17 [59%]), with four (24%) experts selecting anti-HEV IgM alone and one (6%) selecting HEV-RNA alone.

The most controversial diagnostic issue was the use and expected utility of liver biopsy for presumptive severe IIH. The experts were asked under which scenarios they performed a liver biopsy. Eight (47%) of them recommended a liver biopsy for all subjects with severe IIH regardless of prior therapy with corticosteroids, and two (12%) recommended a biopsy, but only prior to initiating the treatment with corticosteroids. The scenario with the highest degree of agreement (11/17 [65%]) on performing a liver biopsy was IIH without improvement after therapy with corticosteroids, as recommended by both the European Society for Medical Oncology (ESMO) and the American Society for Clinical Oncology (ASCO), summarized in Table 2 [4,5]. 

#### 3.1.3. Treatment

Participants were asked about the criteria they used to initiate treatment with corticosteroids in patients with severe IIH. Five (29%) of them favored therapy with corticosteroids for all patients with severe hepatitis. The most common option (10/17 [59%]) was initiating corticosteroid use only for those patients with grade 3 or 4 IIH that does not improve after discontinuation of ICIs, although 2 participants also advocated using corticosteroids in cases with persistence of increased transaminase levels after stopping immunotherapy regardless of the Common Terminology Criteria for Adverse Events (CTCAE) grade of the hepatitis. Only four (24%) respondents based the indication for corticosteroid use on the histologic presence of severe inflammation in liver tissue.

**Figure 1:**
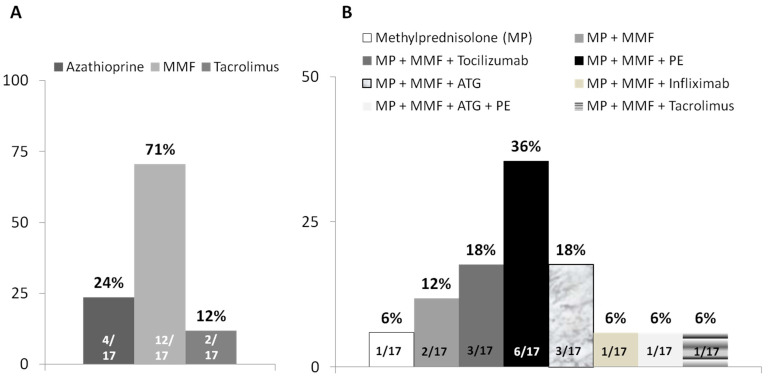
**Treatment Preferences.** (**A**) Preferred drugs in second-line therapy for immune-mediated hepatitis refractory to treatment with corticosteroids. (**B**) Treatments of choice for acute liver injury (defined by increased bilirubin level plus INR > 1.5) owing to immune-mediated hepatitis associated with treatment with ICIs. ATG, anti*-*thymocyte globulin; MMF, Mycophenolate mofetil; MP, methyl-prednisolone; PE, plasma exchange.

The preferred second-line therapy for severe IIH not responding to corticosteroids was mycophenolate mofetil (MMF) (Figure 1A). For acute liver injury owing to IIH, defined as increased values of bilirubin and coagulopathy (International Normalized Ratio, INR >1.5) [8], the most common treatment of choice was a combination of corticosteroids with MMF and plasma exchange (6/17 [35%]), followed by tocilizumab or anti*-t*hymocyte globulin (Figure 1B).

### 3.2. Management of Renal irAEs

#### 3.2.1. Participants

Out of 69 experts who were sent surveys regarding renal irAEs, 20 (29%) responded. The characteristics of the respondents are summarized in Table 2. All but one worked at a university-affiliated hospital and they had a median time of practice of 12 years. Most of the respondents (90%) estimated that they managed at least 10–20 patients with immune-related acute kidney injury (AKI) per year, with half of them providing care for at least 40 patients per year. Most of the respondents worked in the United States, followed by Spain.

#### 3.2.2. Diagnosis

A majority (12/20 [60%]) of the experts agreed that a kidney biopsy should be performed for a suspected irAE in patients having Kidney Disease: Improving Global Outcomes (KDIGO) stage 2 or 3 AKI, unless the injury has a clear alternative etiology and regardless of prior corticosteroid-based therapy. Eight (40%) chose to perform a biopsy in this scenario even prior to the onset of corticosteroid administration. Most of the experts (13/20 [65%]) do not systematically investigate eosinophiluria (urine eosinophils).

With respect to imaging, most of the participants (14/20 [70%]) recommended a renal ultrasound or a computed tomography scan for all ICI-treated patients with AKI. The six other experts only recommended performing imaging for patients with KDIGO stage 2 or 3 AKI.

#### 3.2.3. Treatment

The participants were asked about their use of corticosteroids for immune-related AKI. Some of them voted for more than one choice. Eleven (55%) were in favor of corticosteroid-based therapy for ICI-related AKI in patients with serum creatinine levels twofold to threefold higher than the baseline (as recommended by the ASCO and National Comprehensive Cancer Network guidelines) [5,9]. Additionally, eleven participants were in favor of corticosteroid use in all cases of AKI with a histologic diagnosis of acute tubulointerstitial nephritis in a kidney biopsy. With respect to the duration of corticosteroid-based therapy, 60% of the participants chose tapering over 4–6 weeks, and six of them chose tapering over 12 weeks. About two thirds (65%) of the participants recommended treatment with MMF in steroid-refractory cases. Decisions about a rechallenge with ICIs varied among the experts, with 85% saying they would do so after complete kidney function recovery.

### 3.3. Management of Immune-Related Myositis

#### 3.3.1. Participants

We surveyed members of two groups conducting research in irAEs in rheumatology: the Outcome Measures in Rheumatology immune-related adverse events special interest group [10] and a newly formed consortium in the United States interested in collecting prospective data on cancer patients who experience irAEs with ICI use. All group members surveyed (N = 25) were physicians with clinical expertise in the management of irAEs who practiced in university-affiliated institutions. Nineteen (76%) physicians responded to the survey: 11 in the United States, 5 in Europe, 2 in Canada, and 1 in Australia. Their median number of years in practice was 10. Twelve (63%) respondents reported giving treatment to a minimum of 30 patients with rheumatic irAEs per year.

We presented the participants with two cases of management of myositis. Case 1 (myositis alone) was a 60-year-old male patient who had the recent onset of weakness and myalgia in the upper and lower extremities, was unable to walk or get out of bed on his own, with grade III symptoms as per the CTCAE, had a creatine kinase level of 5000 U/L, and had no evidence of myocarditis or myasthenia gravis. Case 2 (myositis and myocarditis) was a similar patient but with increased troponin I and T levels, imaging evidence of myocarditis, and a normal ejection fraction.

#### 3.3.2. Diagnosis

The participants were asked about whether they would recommend a muscle biopsy for case 1 (only for clinical need, not for research specified in the question), with 11 (58%) saying they would do so. For Case 2, seven (37%) participants would recommend a muscle biopsy and nine (47%) would recommend an endomyocardial muscle biopsy (with some recommending both). Seven (37%) participants would not recommend either biopsy.

#### 3.3.3. Treatment

For case 1 (myositis only), 11 (58%) respondents recommended initiating corticosteroid use orally in doses ranging from 60 mg to 2 mg/kg daily. Three experts recommended intravenous (IV) pulses of corticosteroids ranging from 500 to 1000 mg. Seven (37%) recommended adding concomitant therapy to corticosteroids, most commonly IVIG. For patients in whom initial therapy failed, we observed a poor agreement in subsequent therapies, with varying recommendations that included IV corticosteroid pulses, IVIG, abatacept, interleukin-6 inhibitors, rituximab, MMF, methotrexate, anti-thymocyte globulin, and plasma exchange.

**Figure 2:**
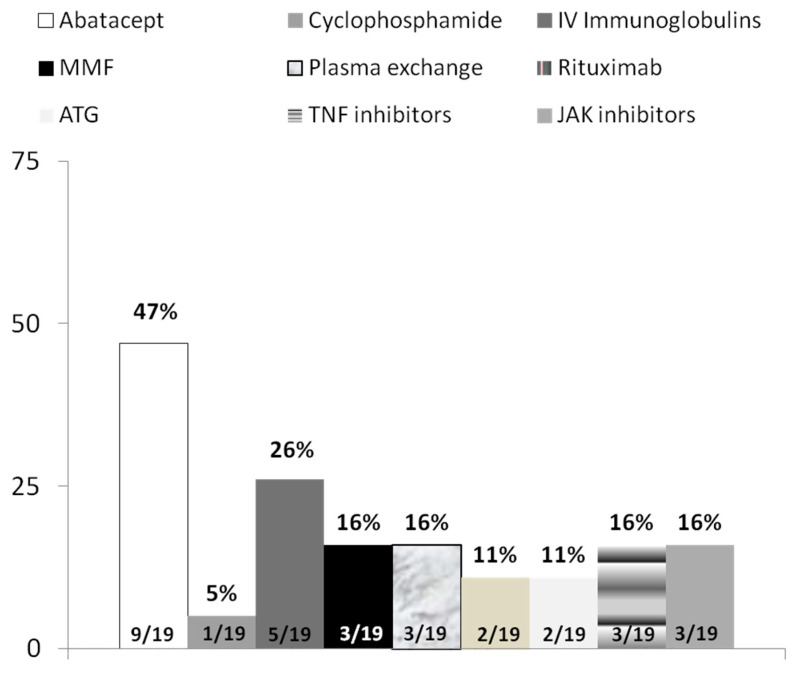
Choice of second-line therapy for a patient with myositis and concomitant myocarditis failing to respond to initial treatment after 2 weeks. ATG, anti-thymocyte globulin; MMF, Mycophenolate mofetil; IV, intravenous; TNF, tumor necrosis factor.

For case 2 (myositis and myocarditis), most of the participants (18/19 [95%]) would initiate treatment with corticosteroids at doses ranging from 1 mg/kg/day to 1000 mg in IV pulses. Fourteen (74%) experts would add concomitant therapy, most commonly with IVIG (53%); three would add abatacept, one would add azathioprine, and one would add a plasma exchange. For patients in whom the initial therapy failed, about half of the participants recommended more than one therapy, the most common being abatacept and IVIG. Other recommended therapies were MMF, Janus kinase inhibitors, rituximab, infliximab, anti-thymocyte globulin, cyclophosphamide, and plasma exchange (Figure 2).

## 4. Discussion

We report the results of a survey of experts in the management of IIH, renal irAEs, and myositis. We chose these irAEs because they can significantly increase morbidity and mortality and often lead to the termination of ICI-based therapy. Several guidelines have addressed therapy for these complications, but they are based primarily on expert opinion, as evidence regarding the comparative efficacy of different immunosuppressants and the potential detrimental effects of these agents on tumor immunity induced by ICIs is scarce. Our aim was not to ascertain the general practice patterns of oncologists or other specialists at large, which we assumed would show large differences, but rather to evaluate how a select group of experts with extensive experience in the field manage these irAEs. Our findings show that even among experts, there is little consensus for important clinical decisions, despite the availability of guidelines, highlighting the need for additional research in this important field. 

Regarding IIH, we observed unanimous agreement by the respondents only for the use of imaging in severe cases. This approach seems appropriate, as real-world data have demonstrated that the most common cause of the elevation of transaminase levels in patients receiving ICIs is liver metastasis of the underlying cancer [11,12]. Furthermore, this practice is in line with the recommendations from both the ESMO and ASCO [4,5]. However, the criteria for the performance of a liver biopsy in IIH patients remain controversial. These guidelines recommend a biopsy in cases without improvement after corticosteroid-based therapy. This was supported by 65% of the respondents, whereas only two participants advocated a universal biopsy prior to the onset of the corticosteroid administration. Differences in preferences for a biopsy may be related to the indication for and timing of the initiation of therapy with corticosteroids.

Real-world data demonstrated that the discontinuation of ICIs is often the first-line intervention for severe IIH [13]. Nevertheless, close to a third of the experts were in favor of initiating corticosteroids for all patients with severe hepatitis, whereas only four experts based their preference for this indication on the findings of liver histology. With respect to second-line therapy, in line with the ESMO and ASCO guidelines [4,5], MMF was the preferred drug for refractory IIH (71% of experts). For patients with acute liver injury, we observed a great deal of heterogeneity with respect to the third-line therapy selected by the experts, with about a third of the respondents opting for plasma exchange, which is the treatment most frequently described in the literature for both severe hepatitis and many life-threatening irAEs [14,15,16,17]. Surprisingly, about 18% of the experts selected tocilizumab, although authors have described its use only in case reports of IIH with associated coagulopathy [18], and its administration is not recommended by the ESMO or ASCO guidelines [4,5]. This preference for tocilizumab may result from the increasing use of interleukin-6 receptor inhibitors for the treatment of other refractory irAEs such as arthritis and pneumonitis [19,20].

In the survey regarding ICI-induced renal irAEs, we did not see a unanimous agreement for any of the questions asked. This may be partly related to a lack of information and the scarcity of published studies in this area. More than half of the experts recommended a kidney biopsy for suspected immune-related AKI in patients who had KDIGO stage 2 or 3 AKI, unless they had a clear alternative etiology regardless of prior corticosteroid use. Additionally, with respect to the diagnosis of renal irAEs, 35% of the respondents were in favor of assessing urine eosinophils. In this respect, previous studies have demonstrated a low sensitivity and specificity for the presence of urine eosinophils in the diagnosis of classical acute interstitial nephritis [21]. 

Responses related to the use of corticosteroids for renal irAE were split, with some in favor of following the ASCO and National Comprehensive Cancer Network clinical practice guidelines, which recommend treatment with corticosteroids for ICI-related AKI in patients with serum creatinine levels twofold to threefold above baseline [5,9], and others in favor of starting corticosteroid-based therapy in all cases of AKI related to ICI use along with biopsy-proven acute tubulointerstitial nephritis. The recommendations and practice patterns for the tapering of corticosteroids varies across studies. A recent retrospective study suggested that the responses of ICI-induced nephritis were similar with rapid and standard corticosteroid tapering, an approach that can limit the secondary adverse events associated with long-term steroid regimens. Lee et al. [22] showed that patients with ICI-induced nephritis have excellent kidney outcomes when given corticosteroids tapered rapidly over 3 weeks. In our survey, about half of the experts favored tapering over 4–6 weeks, whereas 25% selected a longer period of 12 weeks.

Few studies have addressed whether patients with renal irAEs can undergo a rechallenge with ICIs. García-Carro et al. [23] reported an ICI rechallenge in 10 patients. Of these, seven had an improved kidney function with a return to their baseline creatinine when immunotherapy was restarted. Only 2 of the 10 patients had a second AKI episode with the reinitiation of ICI use. Additionally, Gupta et al. [24] recently demonstrated that in 121 patients who underwent an ICI rechallenge, 20 (17%) had recurrent ICI-related AKI. It is of note that survival did not differ between patients who underwent a rechallenge and those who did not. In the present survey, when experts were questioned about whether to reinitiate ICI therapy, the majority of them considered a rechallenge, but only when kidney function was completely recovered.

Rheumatic irAEs, primarily arthralgia, arthritis, and myalgia, are common, whereas myositis is not, developing in less than 1% of cancer patients who receive ICIs [25]. However, immune-related myositis is associated with severe morbidity and mortality, especially when it presents with concomitant myocarditis and/or myasthenia gravis [17,26,27]. Evidence of the effectiveness of the management of myositis in the literature is scarce, with most data coming from small case series. In addition, given the potential severity of this complication, it is often aggressively treated with different agents, which limits comparisons across different interventions. Recommendations for the management of myositis are included in the ESMO and ASCO guidelines, but because of the lack of robust evidence, they are not definite with respect to specific interventions. For instance, the ASCO guidelines state “consider...biopsy on an individual basis when diagnosis is uncertain” [5]. The European League Against Rheumatism has published general considerations for the management of rheumatic irAEs but without systematic guidelines, and their recommendation is to consider biopsy on a case-by-case basis [28].

Our survey demonstrated marked variation in practice patterns for diagnosis and the treatment of myositis across experts. For myositis alone, only 58% of the respondents recommended a muscle biopsy. Similarly, 47% of them recommended an endomyocardial biopsy for myositis with myocarditis. With respect to treatment, for patients with myositis only, the selected treatments varied from 60 mg of corticosteroids daily to IV corticosteroid pulses of 1000 mg. Moreover, about one third recommended adding concomitant therapy to corticosteroids, most commonly IVIG. Recommendations for second-line therapy varied across experts and included synthetic, targeted, and biologic disease-modifying antirheumatic drugs, IVIG, and a plasma exchange. For cases of myositis with myocarditis, most of the experts would initiate treatment with corticosteroids at doses varying from 1 mg/kg/day to IV pulses of 1000 mg. Most (74%) of the experts would initiate concomitant therapy, generally with IVIG. The most common second-line therapy for refractory myositis was IVIG (when not offered initially) or abatacept, a CTLA-4 agonist.

To the best of our knowledge, this is the first survey of specialists who are experts in the management of selected severe irAEs secondary to ICI-based therapy. Our survey had limitations. First, the sample size was small and consisted almost exclusively of academic physicians. Moreover, most of the returned surveys were from the United States, and roughly half of the returned IIH surveys were from Spain. However, although the responses may not reflect general practice, our intent was to survey experts because this is an emerging field with very scarce robust data supporting evidence-based practice. Second, a survey may not necessarily reflect clinical practice, where many other considerations related to the health system, practice patterns of different members of multidisciplinary teams, and preferences of patients and their families influence decisions about management. Finally, many of the survey participants chose more than one answer for some of the questions, suggesting uncertainty about the best course of action.

## 5. Conclusions

In summary, these three surveys suggest substantial differences among experts in practice patterns regarding the diagnosis and treatment of selected severe irAEs. Our findings support the need for robust prospective studies and clinical trials comparing strategies for the management of irAEs induced by therapy with ICIs.

## Figures and Tables

**Table 1:** Characteristics of the Experts Who Completed the Surveys.

	*n* (%)		
Characteristic	IIH	Renal irAEs	Myositis
	(*n* = 17)	(*n* = 20)	(*n*= 19)
**Country**			
Australia	0	0	1 (5)
Belgium	0	1 (5)	0
Canada	1 (6)	0	2 (11)
France	2 (12)	1 (5)	1 (5)
Germany	0	0	2 (11)
Italy	2 (12)	1 (5)	0
Netherlands	0	1 (5)	1 (5)
Poland	0	1 (5)	0
Japan	1 (6)	0	0
Spain	10 (59)	2 (10)	0
United Kingdom	0	0	1 (5)
United States	1 (6)	11 (55)	11 (58)
Taiwan	0	1 (5)	0
Turkey	0	1 (5)	0
**Years in practice**			
<5	0	1 (5)	5 (26)
10-May	6 (35)	6 (30)	3 (16)
20-October	4 (24)	10 (50)	7 (37)
20–30	3 (18)	1 (5)	2 (11)
>30	4 (24)	2 (10)	2 (11)
Estimated number of new patients with irAEs seen per year *			
<10			
19-Oct			
20–29	3 (18)	2 (10)	1 (5) *
30–40	5 (29)	4 (20)	1 (5)
>40	3 (18)	3 (15)	4 (21)
	5 (29)	0	5 (26)
	1 (6)	11 (55)	7 (37)
**University hospital affiliation**	17 (100)	19 (95)	19 (100)

* Only 18 experts responded. The number refers to the patients seen with any rheumatic irAEs rather than only myositis.

**Table 2:** Recommendations for the Performance of Biopsy According to International Oncology Guidelines.

	Recommendation	
Biopsy	ASCO [5]	ESMO [4]
**Liver**	Consider in steroid-refractory cases to rule out other entities	Consider in steroid- and mycophenolate-refractory cases
**Kidney**	Kidney biopsy should be discouraged until steroid-based treatment has been attempted	Grade 2: creatinine level >1.5–3.0 times baseline or >1.5–3.0 times ULN; discuss with nephrologist; early consideration of renal biopsy is helpful, which may negate the need for steroids and determine whether renal deterioration is related to ICIs or other pathology
**Muscle**	Myositis: Consider muscle biopsy on an individual basis when diagnosis is uncertain and overlap with neurologic syndromes such as myasthenia gravis is suspected. Myocarditis: Endomyocardial biopsy should be considered for patients who are unstable, failed initial therapy, or in whom the diagnosis is in doubt	Not specifically discussed; general guidance is to consider tissue biopsy in cases with diagnostic doubt about the etiology of the complication and in whom management would be altered by the outcome of the biopsy procedure

## Data Availability

Available if requested.

## Appendix A

1. **Immune-mediated hepatitis survey**
  - Country:
  - Years at practice:
  - University hospital: Yes/No
  - Estimated number of attended patients with immune-related adverse events

0. <10
1. 10–20
2. 20–30
3. >40

**Answer:**

1. **When do you perform a liver biopsy for suspected immune-related hepatitis?**
  - All grade-3 and grade-4 immune-related hepatitis, prior to the beginning of corticoids
  - All cases of immune-related hepatitis without improvement after therapy with corticoids (guidelines recommendation)
  - All grade-3 and grade-4 immune-related hepatitis, regardless of prior corticoids therapy
  - All grade-3 and grade-4 immune-related hepatitis, except those with severe hepatitis (Bilirubin > 2.5 mg/dL plus INR > 1.5)

**Answers:**

2. **Do you systematically rule out acute hepatitis E in all suspected cases of grade-3 and grade-4 immune-related hepatitis?**
  0. Yes, by means of anti-HEV IgM
  1. Yes, by means of HEV-RNA
  2. Yes, by means of both anti-HEV IgM and HEV RNA.
  3. No.

**Answers:**

3. **Do you recommend an imaging test (liver US or CT scan) in all cases of grade-3 and grade-4 immune-related hepatitis?**
  0. Always.
  1. Never.
  2. Just in case of concomitant increase of AP and/or GGT

**Answers:**

4. **When do you start corticoids for immune-related hepatitis?**
  - All grade-3 and grade-4 immune-related hepatitis
  - Only grade-3 and grade-4 immune-related hepatitis with severe inflammation at the liver biopsy
  - All grade-3 and grade-4 immune-related hepatitis without improvement after temporary discontinuation of ICI.
  - All cases of immune-related hepatitis without improvement after temporary discontinuation of ICI, regardless of the CTCAE grade of hepatitis.

**Answers:**

5. **Which immunosuppressant do you prefer for steroid-refractory immune-related hepatitis:**
  0. Azathioprine
  1. MMF
  2. Tacrolimus
  3. Cyclosporin
  4. Other:

**Answers:**

6. **Which therapy do you use for patients with severe immune-related hepatitis (Bilirubin > 2.5 mg/dL plus INR > 1.5):**
  0. Corticoids + MMF + Tocilizumab
  1. Corticoids + MMF + Plasma exchange
  2. Corticoids + MMF + Antithymocyte globulin
  3. Corticoids + MMF + Infliximab
  4. Others:

**Answers:**

2. **Immune-mediated renal events survey**
  - **Affiliation:**
  - **Country:**
  - **Years at practice:**
  - **University hospital:**
  - **Onconephrology unit or outpatient consult:**
  - **Estimated number of attended patients with immune-related adverse events x Year:**

1. <10
2. 10–20
3. 20–30
4. >40

**Answer:**

1. **When do you perform a kidney biopsy for suspected immune-related AKI?**
  - All cases of ICI-treated patients who develop AKI unless there is a clear alternative etiology for the disease (dehydration, obstruction…) without improvement after therapy with corticoids
  - ICI-treated patients who develop KDIGO stage 2 or 3 AKI unless there is a clear alternative etiology for the AKI, prior to the beginning of corticoids
  - ICI-treated patients who develop KDIGO stage 2 or 3 AKI unless there is a clear alternative etiology for the AKI regardless of prior corticoids therapy
  - ICI-treated patients who develop KDIGO stage 3 AKI unless there is a clear alternative etiology for the AKI

**Answers:**

2. **Do you systematically rule out eosinophiluria (urine eosinophils) in all suspected cases of AKI related ICI?**
  - Yes, always.
  - Yes, sometimes.
  - No.

**Answers:**

3. **Do you systematically recommend a renal US or CT scan in all cases of ICI-treated patients who develop AKI?**
  - Always.
  - Never.
  - Just in case of stage 2 or 3 AKI.

**Answer:**

4. **When do you use corticoids for immune-related AKI?**
  - ICI related AKI with SCreatinine 2–3× baseline (as recommended by ASCO and NCCN clinical practice guidelines)
  - Only KDIGO stage 3 AKI related to ICI with severe inflammation at the kidney biopsy (ATIN)
  - KDIGO stage 2 and 3 AKI related to ICI without improvement after temporary discontinuation of ICI.
  - All cases of AKI related to ICI with ATIN diagnosis in kidney biopsy.

**Answer:**

5. **In case you started steroids how long do you use them?**
  - Steroids tapering over 2 weeks
  - Steroids tapering over 4–6 weeks
  - Steroids tapering over 3 weeks
  - Steroids tapering over 12 weeks
  - Steroids tapering over 24 weeks

**Answer:**

6. **Which immunosuppressive therapy do you prefer for steroid-refractory immune-related AKI.**
  - Azathioprine
  - MMF
  - Tacrolimus
  - Cyclosporin
  - Other

**Answer:**

7. **When do you recommend ICI rechallenge in immune-related AKI patients with cancer disease progression?**
  - Never
  - Rechallenge in patients with CTCAE grade 1 toxicity.
  - Rechallenge in patients with KDIGO stage 1 or 2 AKI with biopsy-proven ICI.
  - Rechallenge after kidney function has completely recovered.

**Answer:**

3. **Immune-mediated myositis survey**

**Country of practice:**

**Years in practice:**

**Affiliated with university or academic center: Yes ____ No____**

**Approximately, how many patients with rheumatic immune-related adverse events do you see per year (mark one only)?**

☐ <10
☐ 10–20
☐ 20–30
☐ 30–40
☐ >40

**irAE- Myositis case. 60 year old patient with recent onset of weakness and myalgia in upper and lower extremities, unable to walk or get out of bed on their own, grade III as per CTCAE, creatine kinase (CK) of 5000 U/L, and no evidence of myocarditis or myasthenia gravis:**

1. Would you recommend a muscle biopsy (for clinical purposes, not research)?
  Yes ____  No____
2. What would be your initial recommendation for treatment with glucocorticoids?
  Route of administration: oral_______ IV_________
  Dose and glucocorticoid agent:__________
3. Would you add any other treatments at this time? Please specify.

**After 2 weeks of treatment, there is only mild improvement, patient remains bedridden, CK is 4000 U/L.**

4. What would be your recommendation for management at this time?________________

**irAE- Myositis with myocarditis. For a 60 year old patient with recent onset of weakness and myalgia in upper and lower extremities, unable to walk or get out of bed on their own, grade III as per CTCAE, creatine kinase (CK) of 5000 U/L, with marked increases in troponin I and T, ejection fraction of 50% and imaging evidence of myocarditis by MRI:**

1. Would you recommend a muscle biopsy (for clinical purposes, not research)?
  Yes ____  No____
2. Would you recommend an endomyocardial muscle biopsy (for clinical purposes, not research)?
  Yes ____  No____
3. What would be your initial recommendation for treatment with glucocorticoids?
  Route of administration: oral_______ IV_________
  Dose and glucocorticoid agent:__________
4. Would you add any other treatments at this time? Please specify.__________________________

**After 2 weeks of treatment, there is only mild improvement, patient remains bedridden, CK is 4000 U/L, and troponins remain high, with ejection fraction at 48%.**

5. What would be your recommendation for management at this time?________________
